# Harnessing beneficial soil bacteria to promote sustainable agriculture and food security: a one health perspective

**DOI:** 10.3389/fmicb.2025.1638553

**Published:** 2025-09-19

**Authors:** Carlos Sabater, Madalina Neacsu, Sylvia H. Duncan

**Affiliations:** Rowett Institute, University of Aberdeen, Foresterhill, Aberdeen, Scotland, United Kingdom

**Keywords:** *Bacillus*, *Paenibacillus*, food security, plant disease, biopesticides, growth hormones, probiotics

## Abstract

Harnessing beneficial soil bacteria for use in agricultural practices offers an exciting and promising pathway to achieving sustainable farming. Soil microbes, including bacteria and fungi, play a pivotal role in nutrient cycling, enhancing soil structure, and promoting plant growth. Certain plant growth-promoting bacteria, such as *Bacillus* and *Paenibacillus* species, are particularly notable for their ability to improve nutrient uptake, suppress pathogens, and enhance plant resilience to environmental stress. By employing these natural bacterial species, farmers can rely less on chemical fertilisers and pesticides, helping the environment and improving soil health. Moreover, soil bacteria may possess potent enzymes systems for breaking down complex carbohydrates, so that the simpler sugars can be used to nourish plants. Genome mining of soil representatives can be used to design novel consortia of soil bacteria (including *Paenibacillus odorifer, P. xylanilyticus* and *Streptococcus cellostaticus*) to cover the maximum number of complementary enzyme activities acting on cellulosic and hemi cellulosic materials. Similarly, the combination of these strains and *Arthobacter humicola* could be of great interest to maximize the metabolisation of lignocellulosic substrates and to reduce and re-valorise food waste from the food production cycle. Soil bacteria play a pivotal role in advancing One Health by mediating interactions across human, animal, and environmental health. Future research and development should focus on optimizing microbial delivery to different soils and also understanding the complex interactions within the soil microbiome to maximize their benefits in diverse farming systems.

## 1 Introduction

In the face of a rapidly changing climate and the urgent need for sustainable agriculture, harnessing beneficial microbiota, including next generation probiotics which to date have mostly been considered for human health (Al-Fakhrany and Elekhnawy, 2024), has emerged as a promising approach to address the problem. There is considerable interest in using next generation probiotics directly for both human health (Abouelela and Helmy, 2024) and environmental health thereby promoting the One Health concept (Harutyunyan et al., 2022). Soil microbial management enhances One Health outcomes by reducing agrochemical inputs, improving crop nutritional quality, and mitigating Antimicrobial Resistance (AMR).

Conventional agricultural techniques, such as chemical fertilisers and pesticides, can clearly help to protect crop plants against pathogens and ensure better crop yield. Chemicals employed in agricultural practices, however, can be harmful to the environment and may cause widespread soil and water pollution (Elumalai et al., 2025). Moreover, to respond to the increasing food demands of a growing global population under climate change and pesticide limitations (Tudi et al., 2021), biological pest control is a highly desirable alternative to conventional pesticides, for both health and ecological reasons. The global population in 2024 was approximately 8.2 billion and is expected to continue to grow over the next few decades to reach 9.9 billion by 2050. Therefore an immediate priority of agriculture is to increase crop production to assure food security, which is to ensure access to sufficient, safe and nutritious food to meet dietary needs for a healthy lifestyle, whilst promoting sustainability (van Dijk et al., 2021). Feeding the future growing World population is therefore a major societal challenge. In particular, there is a need to transition away from using chemical fertilisers.

Indeed, the excessive and prolonged use of chemical fertilisers has significantly degraded soil fertility and reduced crop productivity. While they supply key nutrients like nitrogen, phosphorus, and potassium, overuse disrupts soil ecosystems, leading to acidification, compaction, and nutrient imbalances that impair root development and water retention (Pahalvi et al., 2021). Chemical fertilisers also reduce soil organic matter and microbial diversity, weakening nutrient cycling and plant health (Pahalvi et al., 2021). These changes lower crop resilience and increase vulnerability to pests and diseases. Nutrient runoff further contributes to eutrophication and environmental pollution (Liu et al., 2021). To restore soil health and ensure sustainable productivity, integrated nutrient management and organic amendments are essential (Samanta and Sengupta, 2024). Moreover, as a result of the indiscriminate use of chemical pesticides in current farming practice however, pesticide resistance and considerable environmental harm have occurred worldwide (Zhou et al., 2025). Many developing countries also face low crop productivity in agriculture due to plant health problems (George, 2014). The development of innovative, cost-effective biocontrol strategies therefore could create an important avenue for improving the economic status of farmers, increasing nutrition security, reducing environmental impacts, and minimizing health hazards. In this regard, microbial bio-pesticides which are microorganism-based, low-risk, environmentally friendly agents for managing plant pathogens and pest populations is a promising approach for the future. There is considerable interest therefore in using naturally occurring microorganisms to protect plants from diseases. Soil maintains one of the most diverse ecosystems on Earth (Whitman et al., 1998). Certain bacterial strains may be used due to antimicrobials, increasing levels of plant growth hormones, reducing the spread of antibiotic genes and the reliance on chemical pesticides (Lin et al., 2025). Beneficial bacteria are likely to use several strategies to promote plant growth which may include competing with pathogens for resources such as carbon and nitrogen sources, secreting antimicrobial compounds, and activating the plants defense mechanisms (Kiprotich et al., 2025).

Global political instabilities can also lead to shortages of essential crop fertilisers worldwide and fertiliser prices have soared by 300 percent in 2021 as a result (Agriculture in the United Kingdom, 2022). British farmers alone have faced additional fertiliser costs of £1.45 billion following the conflict in Ukraine (Farmers hit by £1.4b fertiliser bill since Ukraine war with costs ‘set to stay high', 2024). These higher input costs are then passed on to consumers or lead to reduced crop yields contributing to food and nutrition insecurity. This crisis calls for development of solutions to overcome the fertilisers shortage, such as the development of biofertilisers that, offer sustainable alternative to maintain soil fertility and support global food production (Chhabra et al., 2024). Nitrogen-fixing bacteria, along with phosphate and potassium solubilizing bacteria are likely to positively contribute to biofertilisers by helping to increase the Nitrogen, Phosphorous and Potassium (NPK) content of soil.

In a changing climate, the deployment of beneficial bacteria therefore offers a sustainable and eco-friendly approach to plant disease management, contributing to global food security while reducing the environmental impact of agriculture (de Souza Vandenberghe et al., 2017).

Besides, there are significant losses in food along the production chain, with over 30% being lost or wasted annually, accounting for 1.3 billion tons (Vesković, 2025). This food waste occurs at every stage from agricultural production, processing, distribution/retailing to consumption and disposal with almost half of food wastage occurring during agricultural production and processing. This potential food loss results in diminished sources of nutrients which include dietary protein, fiber, vitamins, minerals and important bioactive molecules contributing significantly to increasing the carbon footprint of our food system. Concurrently, the rise in non-communicable diseases is exponential and to some extent is directly related to our diets, such as overconsumption of calories, saturated fats, high refined foods, high in sugar and salt and is concomitant with the under consumption of minerals and dietary fiber (Curioni et al., 2022). Under consumption and inadequate long-term, dietary fiber, can have a negative impact on general health and the immune system and may promote cardiovascular disease, inflammatory conditions and obesity (Deehan et al., 2024).

One way to help tackle food loss and boost the quantity and diversity of dietary nutrients is to revalorize the nutrients and useful bioactives through the food cycle by using specialized bacteria with complex carbohydrate activities such as cellulose, hemicellulose, lignin and tannin degrading bacteria (Durica-Mitic et al., 2018; Glowacki and Martens, 2020). As an example, *Bacillus amyloliquefaciens* has been reported to be an excellent biofertiliser agent helping to improve mineral availability and promote plant growth without harmful side effects (Luo et al., 2022). In this review, we report on the importance of key environmental bacteria, particularly from soil ecosystems, to replace chemical fertilisers with biofertilisers and to help reduce and re-valorise food waste from the food production cycle to help deliver on one of the global sustainable development goals which is to end hunger, achieve food and nutrition security and promote sustainable agriculture, environment and health.

## 2 Sustainable agriculture

Plant growth-promoting microorganisms contribute to plant development through multiple mechanisms, including the solubilisation and mineralization of macro—and micronutrients, synthesis of phytohormones, suppression of phytopathogens, and facilitation of nitrogen assimilation (Kumar et al., 2022). The application of these microorganisms as biofertilisers not only enhances crop productivity and soil quality (Mukhtar et al., 2017) but also aligns with the principles of sustainable agriculture by reducing dependency on synthetic agrochemicals and promoting long-term soil health. Sustainable agricultural practices such as cover cropping, composting, and reduced tillage may also help the natural establishment and stability of beneficial soil microbial communities and thereby reducing reliance only on microbial inputs (Kiprotich et al., 2025).

### 2.1 Soil health

Soil and plant root-associated bacteria are numerous and complex (Trivedi et al., 2020), as with other remarkable ecosystems such as mammalian gut ecosystems. Plants provide a multitude of niches for the growth of a diverse range of microorganisms, including bacteria, fungi, nematodes, and viruses. Culture-independent, high-throughput sequencing has greatly expanded the repertoire of microorganisms known to reside in soil and on plants as well as in the environment (Vandenkoornhuyse et al., 2015; Lundberg et al., 2012). Complex microbial communities contain species from diverse phyla and as with many complex microbial ecosystems, there is likely to be a core mix or consortia of bacteria that are optimal for health and that reproducibly associates with a given plant host across a wide range of environments (Hamonts et al., 2018).

Plants coexist with root-associated bacteria and have evolved the ability to enrich and maintain beneficial microorganisms with related biological functions (Santoyo, 2022) to offer protection from abiotic stresses, such as drought and high salinity in addition to biotic stresses, which includes plant pathogens (Gul et al., 2023). Root-associated bacteria play a crucial role in the interplay between plants and insects, and some species or the whole community in native soil can trigger defense responses in plants to improve their performance against above ground insects. There are many insecticidal microorganisms in soil, such as *Bacillus thuringiensis* (Ma et al., 2023). In addition to these roles, soil microorganisms significantly enhance soil structure through the production of extracellular polysaccharides, contribute to nutrient cycling and organic matter decomposition, and support microbial diversity, all of which are essential for maintaining the physical, chemical, and biological integrity of healthy soils.

### 2.2 Reducing plant disease

Plant pathogens and insects are considered to be of major economic significance and are estimated to markedly reduce the world's annual crop yield (Oerke, 2006). Due to their incredible diversity and adaptability, insects are probably the single most challenging pest to control in agriculture worldwide. Insects do not only cause major damage to agricultural crops as pests but are also vectors of diseases. Since the introduction of synthetic insecticides, their application has made a major contribution to improving food production but are also problematic.

The rapid appearance of resistance to insecticides is a major concern in pest management. Insect pest species of economic importance are pests that are resistant to more than thirty different chemical insecticides and this is no longer unusual (The Arthropod Pesticide Resistance Database and Michigan State University, 2025). Moreover, chemical insecticides are troublesome because of their potentially nocuous effects on the environment and public health.

Conventional methods for managing bacterial, fungal, and nematode-induced plant diseases such as chemical pesticides, soil fumigation, and thermal sterilization have been widely adopted in agriculture but are increasingly recognized for their significant drawbacks. These include environmental contamination, health risks to humans and non-target organisms, and the development of resistant pathogen strains. For instance, repeated use of fungicides and nematicides can disrupt soil microbial communities, degrade soil structure, and reduce biodiversity, ultimately compromising long-term soil health and crop resilience. Moreover, the over-reliance on chemical inputs often leads to diminishing returns and increased production costs, particularly in the face of evolving pathogen resistance. These limitations underscore the urgent need for more sustainable and ecologically sound disease management strategies (Akhtar et al., 2024).

After decades of intensive pesticide application, it has become evident that there is no magical, simple solution to control pests in sustainable agriculture. The integration of many different, complementary approaches of chemical and biological control methods may therefore be needed to solve the diverse and challenging problems with pests. *Bacillus thuringiensis* (Bt) is a spore-forming soil bacterium and the insecticidal organism which is on the market for products for microbial control of insects (Bravo et al., 2011; Sanahuja et al., 2011) is used in both agriculture and forestry.

Eco-friendly strategies for managing bacterial, fungal, and nematode pathogens in plants increasingly rely on biological control agents such as beneficial bacteria, fungi, and nematode-antagonistic organisms. These biocontrol agents suppress pathogens through mechanisms like competition, antibiosis, parasitism, and induction of plant systemic resistance, offering a sustainable alternative to chemical pesticides. Such approaches not only reduce environmental contamination and pesticide resistance but also enhance soil biodiversity and plant health. For instance, the use of *Trichoderma* spp., *Pseudomonas fluorescens*, and Bt has shown effectiveness in controlling a range of soil-borne pathogens while promoting plant growth (Bhat et al., 2023; Maleita et al., 2023; Jaiswal et al., 2022), as discussed further in the next section.

### 2.3 Biopesticides

Biopesticides based on pathogenic microorganisms include bacteria that are specific to a target pest offering an ecologically sound and effective solution to pest problems. Entomopathogenic bacteria belonging to the genus *Bacillus* such as *B. cereus, B. sphaericus, B. subtilis*, and Bt, have been used against various insect pests (Gomis-Cebolla and Berry, 2023). Among these, Bt is one of the most commercially exploited bacteria for insect control. It produces a crystal protein (δ-endotoxin) during bacterial sporulation that can cause lysis of gut cells when consumed by susceptible insects (Jisha et al., 2013). In comparison to synthetic pesticides, Bt spores and parasporal crystals are thought to be safer and more specific. A number of Bt formulations are commercially available in the market. However, the most important threat to the continued efficacy of Bt insecticidal proteins (toxins) is the evolution of resistance in target pests (Jurat-Fuentes et al., 2021). Alteration of toxin binding sites is one of the main mechanisms that cause resistance.

Bt is typically applied as topical sprays and has several advantages over conventional chemical insecticides. The pathogenic activity of this bacterium is specific toward a narrow range of insect species and its application is environmentally sound and harmless to humans and other mammals. The use of Bt as a biological control agent does however have some limitations. The bacterium shows low environmental persistence after topical application, mainly because it is sensitive to solar irradiation as well as to the chemical environment on plant leaves and is not a competitive plant colonizer (Bizzarri and Bishop, 2008; Raymond et al., 2010). The susceptible stages of the pest insects are during the early larval stage, Bt therefore only provides short-term crop protection in the field and requires precise application practices (Bravo et al., 2011). Indirect mechanisms by which Bt could inhibit plant pathogens and promote plant growth development includes the production of antimicrobials such as bacteriocins, and enzymes such as chitinases (Kumar et al., 2021; Vojnovic et al., 2024).

Other bacterial strains of *Chromobacterium, Pseudomonas, Serratia*, and *Streptomyces* species may also be active against various insect pests, primarily against lepidopteran caterpillars (Tomar et al., 2024). Most entomopathogenic bacteria produce a variety of toxins with similar mechanisms of action to Bt and therefore, there is a rigorous need to explore novel bacterial isolates having insecticidal potential. Members of the genus *Pseudomonas* are widely distributed in the environment and have been isolated most commonly from insect pests and soil samples (Sarkhandia et al., 2023). Several *Pseudomonas* species such as *P. fluorescens* and *P. putida* are known to have insecticidal properties against many insect pests. Toxins (Fit toxin, Exotoxin A, ExoS, hydrogen cyanide, rhizotoxins) associated with *Pseudomonas* species including *P. aeruginosa*, contribute to pathogenicity by causing sepsis and eventually death of larvae in various insect pests. Pathogenicity of *Pseudomonas* species against insects may also be attributed to hydrolytic enzymes such as proteases, chitinases and phospholipases which are known to be produced by these bacterial strains (Teoh et al., 2021). Metalloproteinases that degrade the internal peptide bonds of proteins inside the gut play a predominant role as a virulence factor of *P. aeruginosa* (Ghssein and Ezzeddine, 2022).

## 3 Complex carbohydrate activities

### 3.1 Genome mining of soil representatives

**Figure 1:**
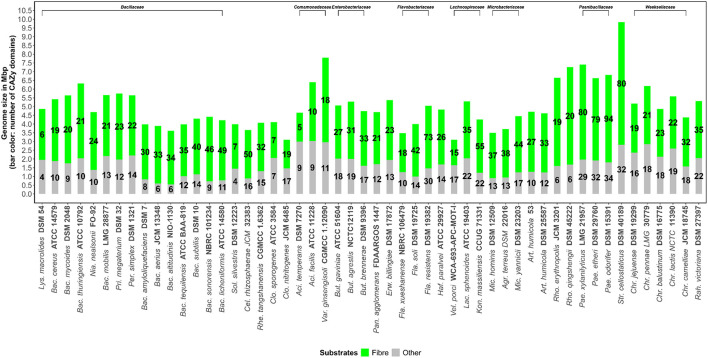
Total number of Carbohydrate-Active enZYmes (CAZymes) acting on fiber (**Fiber**, defined as the sum of pectin, xylan/arabinoxylan, mannan, glucan/xyloglucan, fructan, lignocellulosic materials, resistant starch and malto-oligosaccharides) and other carbohydrate substrates (**Other**) determined in the genome sequences of bacterial soil strains.

There are many mechanisms through which bacteria contribute to soil health which includes breakdown of complex carbohydrate structures. Assessment of the key soil bacterial enzyme complements that have a role in metabolism of such structures were analyzed using the type strains of genomes of representative soil bacterial isolates. Publicly available reference genome sequences of these bacteria were retrieved from the Biotechnology (NCBI) Assembly database (accession codes GCA_000007825.1, GCA_000008425.1, GCA_000196615.1, GCA_000196735.1, GCA_000422705.1, GCA_000685115.1, GCA_000758725.1, GCA_000832985.1, GCA_001012825.2, GCA_001281525.1, GCA_001513965.1, GCA_001592005.1, GCA_001592125.1, GCA_001646745.1, GCA_001654835.1, GCA_001654925.1, GCA_001655005.1, GCA_001884045.1, GCA_002119445.1, GCA_002243645.1, GCA_002770595.1, GCA_002835805.1, GCA_002899875.1, GCA_003385515.1, GCA_003470205.1, GCA_003990875.1, GCA_004330295.1, GCA_006716905.1, GCA_009696045.1, GCA_009749465.1, GCA_013359935.1, GCA_014645135.1, GCA_015278355.1, GCA_019048385.1, GCA_022803015.1, GCA_023913775.1, GCA_029024805.1, GCA_029537415.1, GCA_029894105.1, GCA_030486595.1, GCA_031317525.1, GCA_039521545.1, GCA_039531445.1, GCA_900094985.1, GCA_900100075.1, GCA_900105615.1, GCA_900112975.1, GCA_900168205.1, GCA_900182645.1, GCA_900184995.1, GCA_900446255.1) and proGenomes Database (accession code CP014609.1). These soil isolates belong to fifty two different type strains, which in turn belong to twenty seven different genera and more than twelve different families/phyla. Of these type strains, eleven of the genomes analyzed belong to the *Bacillus* genus which is well recognized as bacteria found in soil ecosystems. Other soil bacteria representatives selected for genome mining include *Acidovorax, Arthrobacter, Buttiauxella, Chryseobacterium, Microbacterium, Paenibacillus* and *Rhodococcus* species. These taxa are commonly found in the soil microbiome and comprise major representatives of nodules endophytic bacteria (Hnini and Aurag, 2024). For the purpose of this study, microbial groups with potential undesirable traits were not included in the comparative analysis. To investigate the metabolic activities of these soil strains, complete Carbohydrate-Active enzymes (CAZymes) profiles found in their genome sequences were annotated (Figure 1). For this purpose, “run_dbcan” software (Zhang et al., 2018), which maps the genome sequences against the CAZy database (http://www.cazy.org/last accessed: 01/05/2025), was used to annotate different CAZymes families. This computational pipeline integrates HMMER software for biosequence analysis to map microbial sequences against CAZy database (dbCAN version 3). This is an expert-curated database of profile hidden Markov models representing the signature domains of CAZyme families. Therefore, run_dbcan software allows functional domain annotation of bacterial CAZymes and assigns specific codes corresponding to the CAZy family of each enzyme (GH, glycoside hydrolase; CE, carbohydrate esterase; PL, polysaccharide lyase; CBM, carbohydrate-binding module; AA, auxiliary activity). To ensure the quality of the data generated, only enzyme domains showing coverage values higher than 0.8 were chosen. Soil strains were grouped according to the CAZymes domains found in their genomes through hierarchical clustering considering a Euclidean distance metric, defined as the distance between two points in Euclidean space (i.e., square root of the sum of the square differences). Then, heatmaps illustrating the presence and absence of different CAZymes were generated. For the purpose of this research, we focused on those CAZymes acting on cellulosic, hemicellulosic (xylan, arabinoxylan) and lignin structures as these are the main carbohydrate structures and complex organic polymers found in the cell walls of plants and the organic matter in soil. Enzyme heatmaps and cluster analysis were performed on R (v.4.4.1).

The total number of CAZymes acting on fiber structures versus genome size is shown in Figure 1. Compared to bacteria from other ecosystems, such as the gut ecosystem, soil bacteria tend to have larger genomes but are variable in size, ranging from around 3-10 Mb (Figure 1). The genomes of almost all soil bacteria are likely to fall within this range. This wide range in genome size is likely to suggest that certain soil bacterial species carry out rather specialist roles whilst others are generalists and have a role in wide ranging activities. Moreover, bacterial strains also have a remarkable range of genes encoding for fiber degradation, which ranges from 5 to 94 genes (Figure 1). Interestingly *Paenibacillus odorifer* DSM 15391*, P. xylanilyticus* LMG 21957 and *Streptococcus cellostaticus* DSM 40189 showed the highest number of CAZymes acting on fiber (defined as the sum of pectin, xylan/arabinoxylan, mannan, glucan/xyloglucan, fructan, lignocellulosic materials, resistant starch, and malto-oligosaccharides).

### 3.2 Soil bacteria with cellulase and hemicellulase (xylan) activities

With respect to metabolism of complex carbohydrates, a certain amount will come from plant cell walls. These are mainly comprised of cellulose which makes up around 30 to 50% of dry weight of plant cell walls and hemicellulose (20-30% dry weight plant cell walls), with usually lower levels of pectin (Zhang et al., 2021). Cellulose is comprised of long chains of β-1,4 linked glucose residues and is unbranched. These long chains form microfibrils through hydrogen bonding and these microfibrils contribute to the rigidity of plant cell walls. Hemicellulose is branched and tends to be of shorter chain length of sugar residues than cellulose and is composed of various sugars, including xylose, mannose, and glucose. Hemicellulose is less crystalline and is usually more easily hydrolyzed by carbohydrate degrading enzymes.

**Figure 2:**
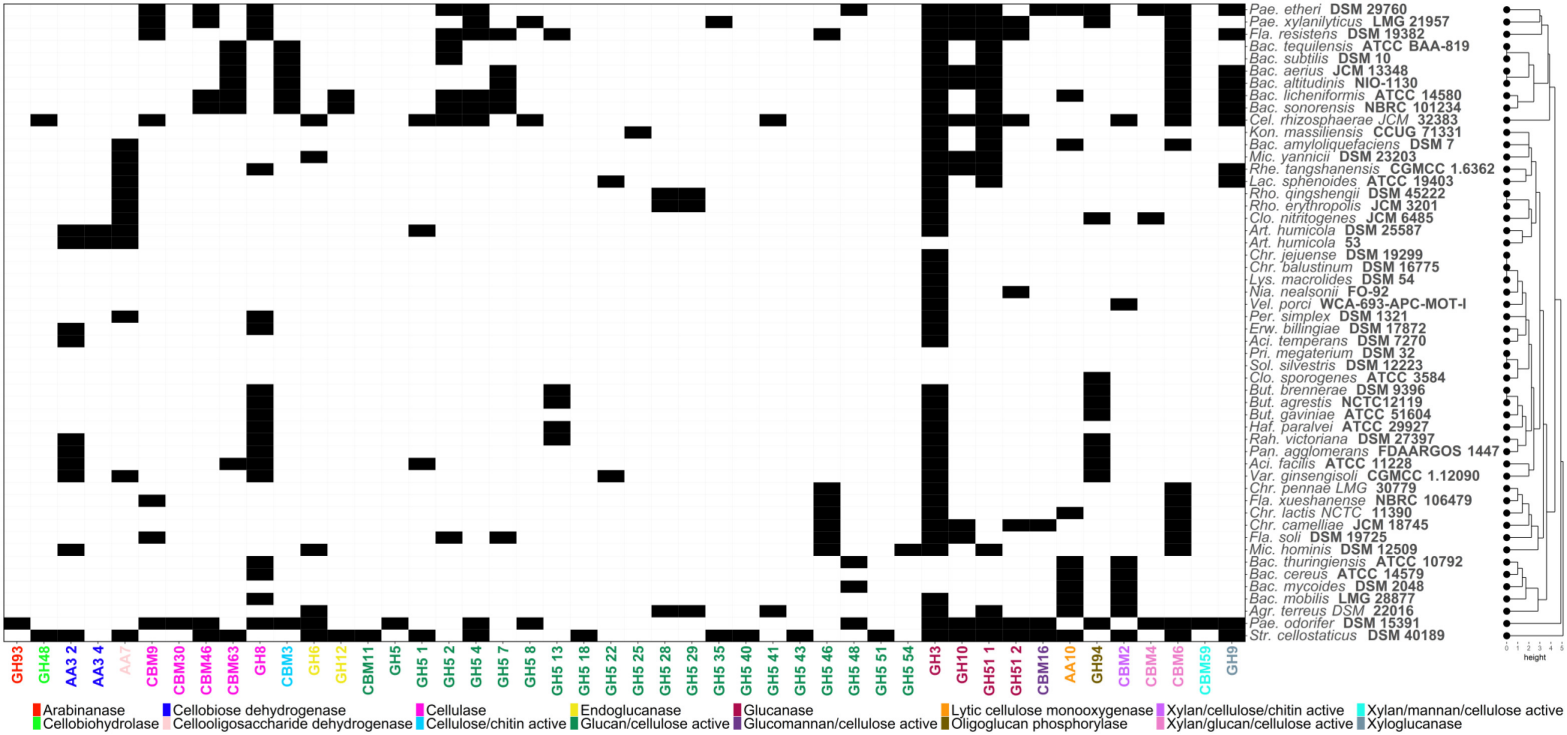
Heatmap showing the distribution of Carbohydrate-Active enZYmes (CAZymes) acting on cellulosic structures. Codes corresponding to the CAZy family of each enzyme have been assigned. Black and white cells indicate presence/absence of each CAZyme, respectively.

Several bacterial species, including those belonging to *Bacillus* and *Paenibacillus* species are recognized for their ability to decompose organic matter in soil. These bacteria therefore are crucial to carbon cycling as cellulose is a major component of plant biomass and is essential for nutrient recycling and soil fertility. The distribution of enzyme activities involved in the metabolisation of cellulosic materials among different soil bacteria is illustrated in Figure 2. For this purpose, CAZymes profiles were compared through hierarchical clustering using R (v.4.4.1) basic function “hclust”. The key enzyme functions required for crystalline cellulose metabolism is an endo—β-1, 4-glucanase that randomly cleaves internal bonds within the cellulose chain. A key enzyme for this process is glycosyl-hydrolase (GH) 5 which was found to be present in the genome of *P. odorifer* DSM 15391 (Figure 2). This bacterium had genes for many of the activities needed to hydrolyze cellulose, as did *S. cellostaticus* DSM 40189 (Figure 2). The action of GH5 then creates new chain ends which are available for further breakdown by exo-glucanases by catalytic modules belonging to families 6, 7, 9, 48, and 74 glycoside hydrolases with the resultant release of cellobiose and glucose. Cellobiose is then cleaved by β*-*glucosidases into glucose (Ahmed et al., 2017). Other enzymes can also increase the efficiency of cellulose degradation including cellobiose dehydrogenases annotated in the genome sequence of *S. cellostaticus* DSM 40189.

**Figure 3:**
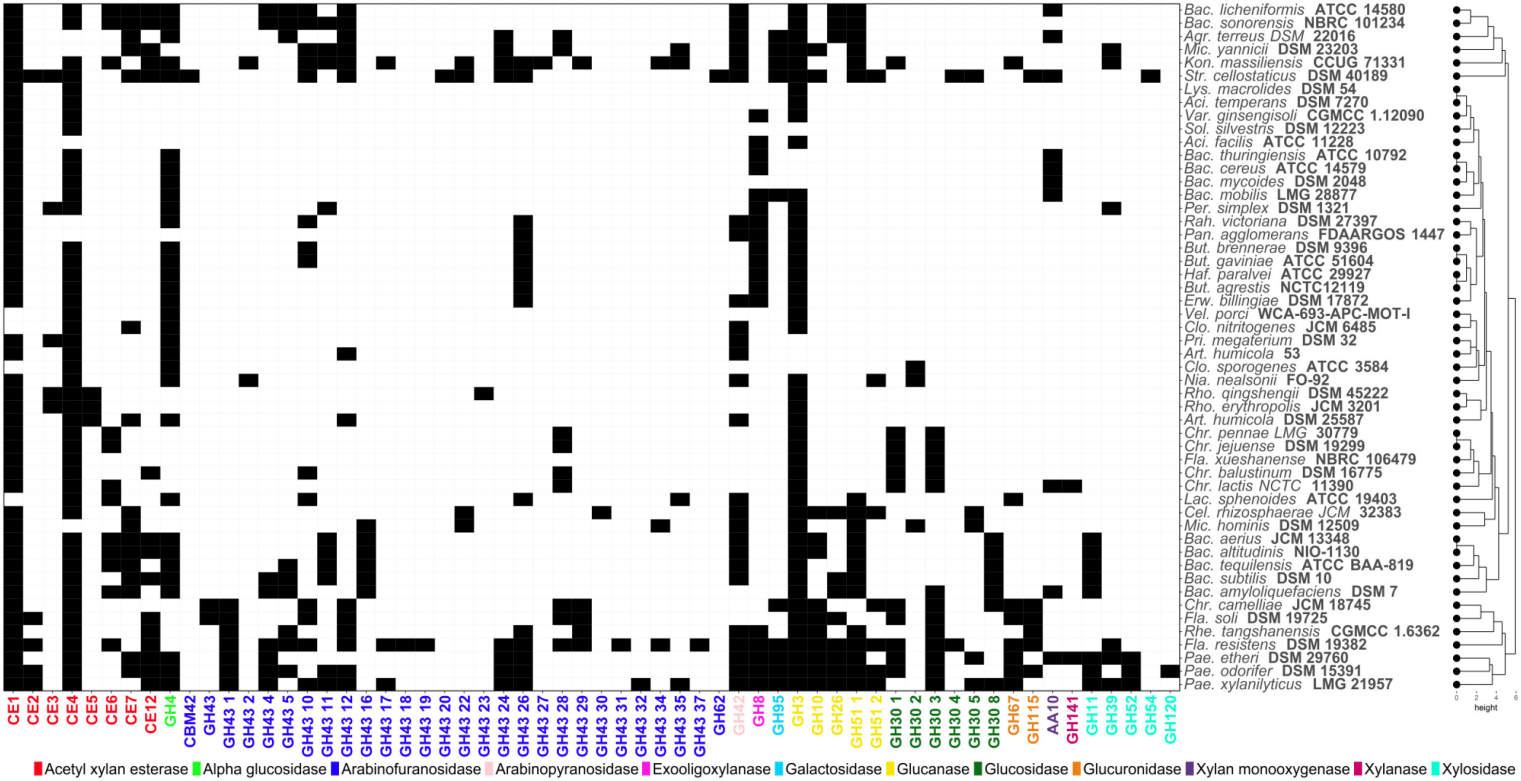
Heatmap showing the distribution of Carbohydrate-Active enZYmes (CAZymes) acting on hemicellulosic (xylan, arabinoxylan) structures. Codes corresponding to the CAZy family of each enzyme have been assigned. Black and white cells indicate presence/absence of each CAZyme, respectively.

Key enzymes involved in hemicellulose breakdown are shown in Figure 3. These enzymes include exo-oligoxylanases (GH8), xylosidases (GH11, GH39, GH52, GH54 and GH120), arabinofuranosidases (GH43) and acetyl xylan esterases (CE - CE7, CE12) (Figure 3). Strains of *Paenibacillus* species including *P. odorifer* DSM 15391 and *P. xylanilyticus* LMG 21957 possess the widest range of GHs involved in hemicellulose hydrolysis compared to other soil bacteria.

Interestingly, the combination of three microbial strains (*P. odorifer* DSM 15391, *P. xylanilyticus* LMG 21957 and *S. cellostaticus* DSM 40189) are likely to cover the maximum number of complementary activities acting on cellulose and hemicellulose.

### 3.3 Soil bacteria with lignin degrading activities

Lignin is also a complex organic polymer found in the cell walls of plants, particularly in wood and bark. The composition can vary significantly depending on the plant species but it is primarily made up of three main monolignol precursors which are coniferyl, p-coumaryl and sinapyl alcohols in varying ratios in different plant species (Liu et al., 2018). These monolignols undergo oxidative coupling to form a three-dimensional network of interlinked aromatic rings which contributes to the integrity of lignin and its resistance to degradation. Lignin plays several crucial roles in plants such as providing structural support and also helps in the efficient transport of water and nutrients in addition to acting as a barrier against microbial attacks (Liu et al., 2018).

Certain fungi and bacteria may be efficient at degrading lignin. Bacteria reported to have a role in degrading lignin include *Pseudomonas, Bacillus* and *Rhodococcus* species (Grgas et al., 2023). The latter are effective in breaking down complex organic compounds including lignin.

**Figure 4:**
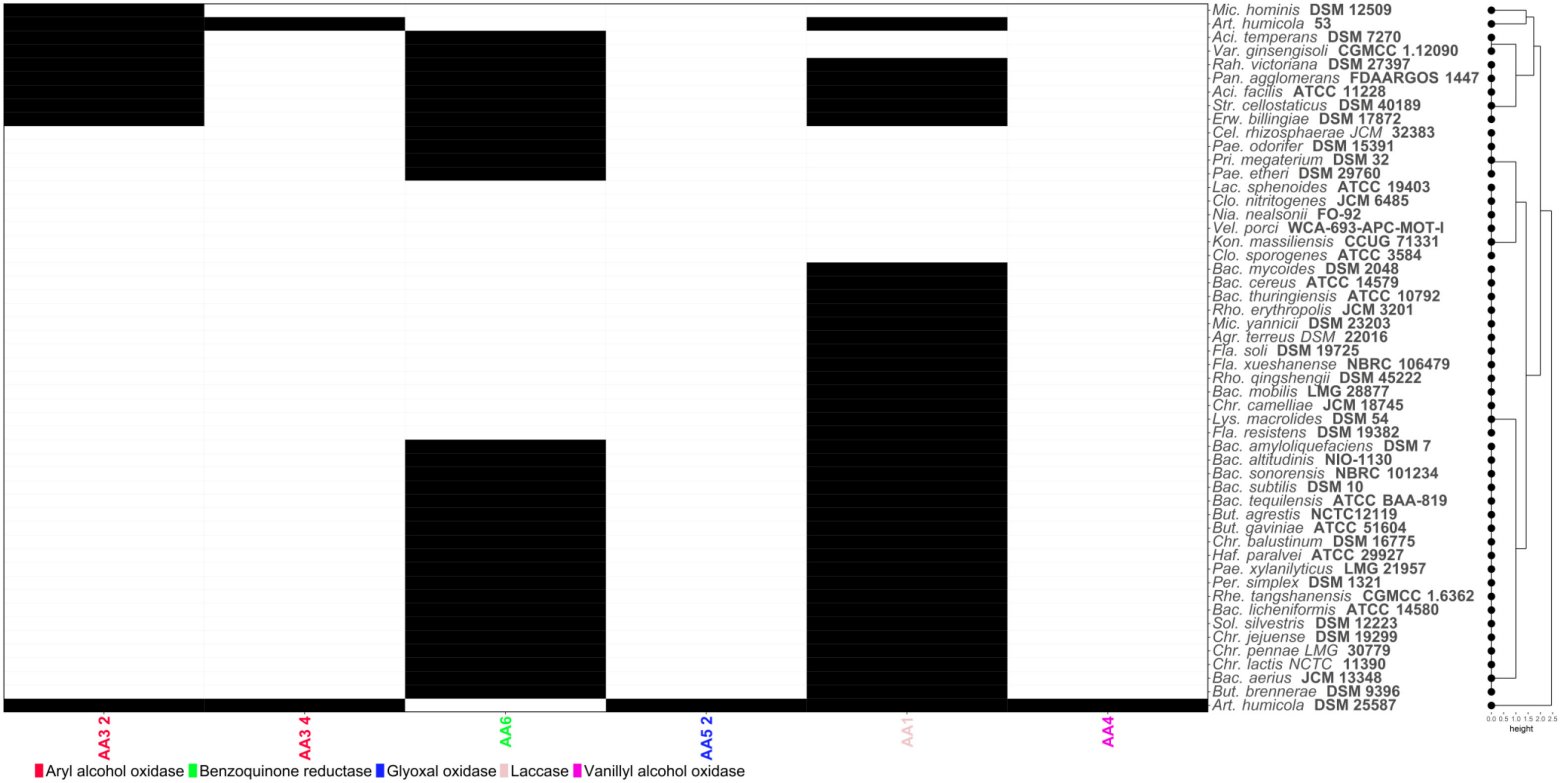
Heatmap showing the distribution of Carbohydrate-Active enZYmes (CAZymes) acting on lignin structures. Codes corresponding to the CAZy family of each enzyme have been assigned. Black and white cells indicate presence/absence of each CAZyme, respectively.

The degradation of lignin typically involves several key enzymes including lignin peroxidases which catalyze the oxidation of lignin, breaking down its complex structure. Manganese peroxidases play a crucial role in the oxidative degradation of lignin by using manganese ions. Laccases are multicopper oxidases and are enzymes that play a role in lignin degradation. Other enzymes such as dioxygenases also have a role in the oxidative cleavage of the aromatic rings in lignin (Kato et al., 2024). To illustrate metabolic differences of common soil bacteria, mining fifty two bacterial genomes revealed that *Arthobacter humicola* DSM 25587 possessed most of the genes for the enzyme families required to degrade lignin including aryl alcohol oxidases, glyoxal oxidases, laccases and vanillyl alcohol oxidases (Figure 4). However, benzoquinone reductase activity involved in the biodegradation of aromatic compounds could not be annotated in the genome sequence of this strain. In contrast, this activity was found in other soil bacteria including promising cellulose and hemicellulose-degrading strains (*P. odorifer* DSM 15391, *P. xylanilyticus* LMG 21957 and *S. cellostaticus* DSM 40189). Therefore, lignin degradation may be most efficient using a consortium of bacterial strains including *A. humicola* DSM 25587 and *Paenibacillus* or *Streptococcus* strains to maximize the number of microbial enzyme activities involved in the metabolism of lignocellulosic substrates.

### 3.4 Soil bacteria with tannase activities

Tannins are secondary metabolites that represent the fourth most abundant plant constituent, after cellulose, hemicellulose, and lignin. Structurally, this group of phenolic compounds could be divided into hydrolysable and condensed tannins (Iqbal and Poór, 2025). Hydrolysable tannins are comprised of a polyol carbohydrate core (usually D-glucose) esterified to phenolic acids such as gallic acid or ellagic acid, forming gallotannins and ellagitannins, respectively. Tannins are generally considered recalcitrant to biodegradation. However, despite their toxic effects on various organisms, some microorganisms have evolved to use gallotannins as carbon and energy sources for growth by the action of a family of enzymes, the tannin acyl hydrolases, commonly known as tannases (de las Rivas et al., 2019).

Tannase, the most studied enzyme in tannin biodegradation, catalyzes the hydrolysis of ester bonds in varied substrates like gallotannins, gallic acid esters, epigallocatechin gallate, and epicatechin gallate leading to the release of gallic acid and glucose. The amino acid sequence analysis of bacterial, yeast, and fungal tannases showed that although the enzymes display divergent sequences, they share a common pentapeptide active site motif of Gly-X-Ser-X-Gly, a typical feature for serine hydrolases. Tannase-producing bacteria have been isolated from a wide variety of sources such as soil, wastewater, compost, forest litter, and feces (de las Rivas et al., 2019). Tannase activity has been reported in a wide range of microbial groups (Tang et al., 2025). Bacterial species that have been confirmed to have tannase production capacity include *Bacillus* species including *B. licheniformis* (Jana et al., 2012) and *B. subtilis* (Jana et al., 2013), *Lactobacillus pentosus* (Kanpiengjai et al., 2019), *Enterobacter cloacae* (Beniwal et al., 2013), *Klebsiella pneumoniae* (Kumar et al., 2015), *Pseudomonas aeruginosa* (Selwal et al., 2010), *Staphylococcus lugdunensis* (Chaitanyakumar and Anbalagan, 2016). On the other hand, several fungal species show tannase activity. The majority of these species belong to *Aspergillus* and *Penicillium* (Tang et al., 2025), including *A. niger* (Sharma et al., 2014), *A. awamori* (Tang et al., 2025), *P. verrucosum* (Bhoite and Murthy, 2015) and *Rhodosporidium diobovatum* (Pan et al., 2020).

## 4 Bacterial nitrogen fixation and phosphatase activities

Nitrogen fixation is an important ecological process supported by bacteria, including *Bacillus* strains. This process is critical to the Earth's nitrogen cycle because it turns atmospheric nitrogen (N) into physiologically useful ammonia (NH_3_) or ammonium ions (NH^4+^). *Bacillus* species with nitrogen-fixing properties, such as *Bacillus megaterium* and *B. polymyxa*, make major contributions to soil fertility and plant growth (Grzyb et al., 2021). These *Bacillus* bacteria have nitrogenases, which, during nitrogen fixation, catalyze the transformation of N gas into ammonia. Plants may directly absorb this ammonia, making it a key source of nitrogen for their growth and development.

Soil bacteria also play a crucial role in nutrient cycling, and those with phosphatase activity are particularly important for releasing phosphorus (P) from organic compounds, making it available for plants (Xie et al., 2024). This includes *Bacillus* species which are well-known for their ability to solubilize phosphorus. Others belong to the *Pseudomonas* and *Burkholderia* genera and these bacterial species are versatile and can thrive in various environments. *Acinetobacter* species are also involved in P solubilisation. *Rhizobium* species, although commonly associated with nitrogen fixation in legume root nodules, some *Rhizobium* strains also possess phosphatase activity. *Bacillus* solubilizes phosphates via secreting enzymes called phosphatases. The activity of these bacteria depends on various factors, including soil P availability, plant species, and microbial communities (Oliverio et al., 2020).

## 5 Soil bacteria producers of plant growth hormones including indole acetic acid

The presence of bacteria in the soil and plant tissues can alter the levels of phytohormones, leading to changes in plant growth, development, and the ability to cope with stress (Egamberdieva et al., 2017). Numerous plant growth-promoting bacteria have been found to possess the capability of synthesizing or degrading certain plant phytohormones. Plant physiological activities are regulated by the action of several different phytohormones including cytokinin, gibberellin, abscisic acid, salicylic acid, jasmonic acid, brassinosteroids, auxin, and ethylene (Gasperini and Howe, 2024).

Plant growth promoting bacteria synthesized auxin is the major mechanism that bacteria use to facilitate plant growth (Glick, 2012; Ludwig-Muller, 2015; Shokri and Emtiazi, 2010; Chudiwal and Nalawade, 2024). Determining the most important hormone for different plants depends on the specific context of plant growth and development. For instance, IAA is crucial for root development, while gibberellic acid is key for stem elongation and seed germination, and cytokinins are vital for cell division and shoot growth (Castro-Camba et al., 2022). Indole Acetic Acid (IAA) promotes a wide range of plant growth traits, including both root and shoot growth, cell expansion, root bacterial colonization, differentiation of vascular tissues, defense against pathogens, stimulation of cell division, elongation of stems and roots and loosening of root cell walls (Mishra et al., 2023). Different plant tissues respond optimally to different IAA concentrations. The IAA is often found in plants in a conjugated (and inactive) form with this conjugated form typically comprising approximately 75% of the total IAA within a plant. Moreover, based on a combination of biochemical and genetic studies, there are at least five separate metabolic pathways for the synthesis of IAA that are found in various bacterial strains which can overlap with one another. The production of IAA has been detected in various genera of plant growth promoting bacteria including *Acetobacter, Acinetobacter, Azospirillum, Arthrobacter, Azotobacter, Bacillus, Bradyrhizobium, Burkholderia, Paenibacillus, Pantoea, Pseudomonas, Rhizobium, Rhodococcus, Serratia*, and *Streptomyces* (Mohite, 2013). Several of these genera including *Pseudomonas* certain species are considered as opportunistic pathogens and therefore are unlikely to be considered for probiotic development (Li et al., 2023).

## 6 Delivery approaches for probiotics for plants

There is an increasing need to develop beneficial single strains and microbial consortia to significantly enhance plant health and growth through helping plants to absorb nutrients and increase resistance to pests and diseases (Chakraborty and Ramteke, 2023). Developing probiotics for plants is an interesting tactic, as certain probiotics may increase plant nutrient (Chakraborty and Ramteke, 2023) absorption such as phosphorus, such as though bacterial phosphatases, in addition to nitrogen more efficiently. Moreover, these beneficial bacteria may enhance root development, leading to stronger and healthier plants whilst other bacteria may help plants cope with environmental stresses such as drought and salinity. Approaches that need to be developed should be low-cost and scalable production of probiotic microbes. Bacterial growth medium can be expensive, therefore low-cost alternatives using agricultural waste products may help minimize production costs. The long-term storage of probiotics also needs to be considered and where possible may include the use of spore forming bacilli. Another consideration is the delivery of probiotics, which may include the addition of probiotic-rich compost or biofertilisers to the soil and/or coating seeds with beneficial bacteria before planting. Alternative approaches include applying probiotics directly to leaves or dipping plant roots in probiotic solutions before transplanting.

## 7 Molecular insights and biotechnological advances in biofertilisers

Certain microbial species have genes that encode a variety of plant growth-promoting properties, such as the production of phytohormones, antimicrobial substances, and enzymes involved in nutrient acquisition. Understanding the genetics of these characteristics will aid in the intelligent development of, for example, *Bacillus* strains with improved ability for various plant-beneficial traits (Hashem et al., 2019). Certain *Bacillus* strains have genes that are involved in nutrient mobilization and root colonization whilst signals generated from these soil bacteria may also stimulate plant defense mechanisms and stress tolerance (Pantigoso et al., 2022).

Recent advances in omics technologies, which incorporate genomics, transcriptomics, proteomics, and metabolomics are revolutionizing biofertiliser development by uncovering microbial genes, regulatory networks, and metabolic pathways that support nutrient cycling, stress tolerance, and plant-microbe interactions (Jain et al., 2024). These insights enable the use of genetic engineering, synthetic biology, and CRISPR-based genome editing to create microbial strains with enhanced traits like improved colonization and multifunctionality. Additionally, biotechnological innovations are facilitating the design of stress-resilient microbial consortia that synergistically promote plant growth and soil health under challenging conditions, paving the way for precision agriculture and sustainable crop production.

Biotechnology, molecular biology, and nanotechnology are however increasingly converging to enhance the role of soil bacteria in promoting One Health. Biotechnology enables the genetic engineering of beneficial microbes to improve traits such as pollutant degradation and plant growth promotion. Molecular biology tools like metagenomics and qPCR provide insights into microbial diversity, function, and interactions, allowing for precise monitoring and optimization of soil health interventions. Complementing these, nanotechnology offers innovative delivery systems, such as nano-encapsulation, to protect and enhance microbial viability in the field, and nanosensors for real-time soil health monitoring. Together, these disciplines support sustainable, data-driven strategies that link environmental, plant, animal, and human health.

## 8 Discussion and conclusions

**Figure 5:**
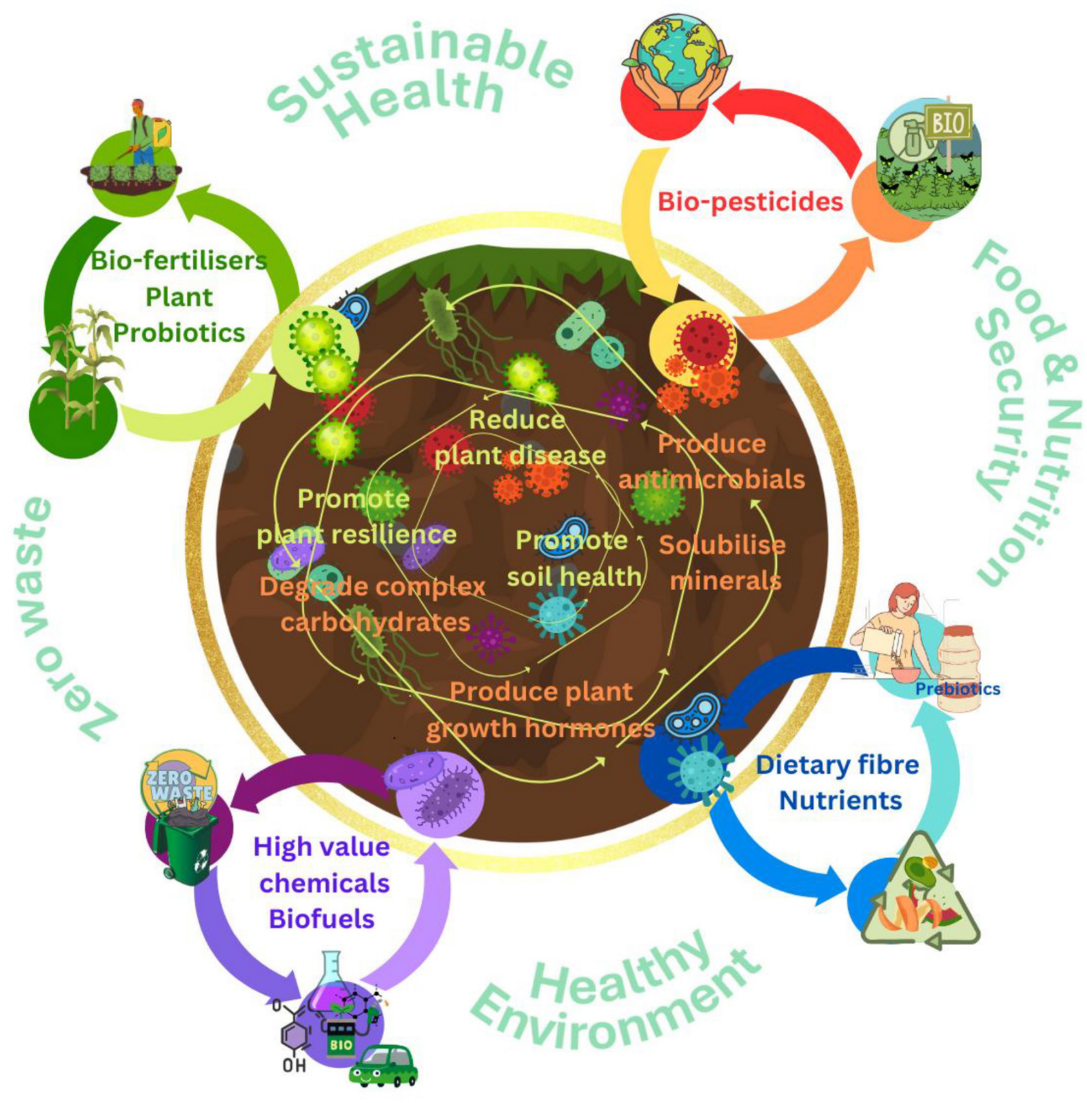
Approaches to harnessing beneficial soil bacteria for promotion of food and nutrition security, sustainable agriculture, and a healthier environment.

Plants have developed a range of different mechanisms to respond to environmental stresses which include, both abiotic factors (e.g., salinity, drought, submergence, temperature) and also biotic factors, such as pathogen infections, using phytohormones. It is evident that, based on all current evidence, the phytohormones produced by plant growth promoting bacteria play an essential role in helping plants to tolerate certain stressful environmental factors. The use of nanotechnology and biotechnology to help deliver beneficial microorganisms or nanoscale materials such as key minerals offer an exciting prospect to improve soil quality, enhance nutrient efficiency and addressing the adverse environmental impact of chemical fertilisers (Arora et al., 2024; El Gharrak et al., 2022). Moreover, nanocarriers can provide a slow and sustained release of nutrients and bacteria over time, reducing the need for frequent applications. Key representatives of the soil microbiota could therefore be harnessed to develop tangible solutions to deliver a more sustainable and resilient agricultural system to promote food security (Figure 5).

Pant—soil microbiome is essential for plant development, soil fertility and crop productivity, but it is severely affected by climate conditions. Soil microbiota plays a crucial role in manipulating the hormonal signaling of plants to manage biotic and abiotic stress. In this context, targeted microbiota-based solutions for precision agriculture could potentially restore soil wellness, fertility, and yield (Muhammad et al., 2025). Modern approaches to optimize plant–microbe interactions and to enhance agricultural sustainability include the identification of plant growth promoting microbes to design field trials. Modern approaches to improve soil vitality and agricultural output include the application of culturomics, metabolic fingerprinting, metagenomics and bioinformatics (Clagnan et al., 2024). Microbiota-based field trials have shifted from single-strain inoculation to multi-species consortia. These microbial consortia include synthetic microbial communities with beneficial and complementary functions as well as complex natural communities from environmental samples. The coupling of culturomics and metabolic fingerprinting can be of great interest to identify new soil health indicators and to discover novel enzymes with biotechnological applications in samples from extreme environments. On the other hand, recent advances in metagenomics and bioinformatics allow the study of unculturable soil bacteria and their metabolic traits including nutrient acquisition mechanisms, protein secretion systems, secretion of primary metabolites and phytohormones, and the presence antibiotic resistance genes (Clagnan et al., 2024). These techniques are of great importance to understand the ecological roles and functionalities of soil bacteria and to tailor microbiome-based formulations to improve soil health under changing climate conditions.

In conclusion, therefore, soil bacteria play a pivotal role in advancing One Health by mediating interactions across human, animal, and environmental health. Several microbial mechanisms underpin this including bioremediation by key bacterial genera which includes *Bacillus* species that can facilitate the degradation of environmental pollutants, including hydrocarbons and heavy metal, thereby enhancing soil and water quality. In agricultural systems, plant growth-promoting bacteria could improve nutrient acquisition, plant growth hormone synthesis, and the suppression of plant pathogens. Collectively these bacterial species are likely to play a key role in supporting food security. Furthermore, soil bacteria are a good source of antimicrobial compounds, some of which are novel, offering potential solutions to the growing threat of antibiotic resistance. Soil bacteria may also have a role in disease suppression via competitive exclusion contributing to the control of both zoonotic and plant disease.

Despite these promising attributes, several limitations constrain the widespread use of soil bacteria to support One Health. Firstly, microbial efficacy is highly sensitive to abiotic stressors such as temperature, pH, salinity, and moisture, which can significantly impair survival and function. Differences in soil structure and conditions further complicates predictions of microbial behavior across diverse field conditions. Secondly, many beneficial bacterial strains exhibit ecosystem specificity, limiting their effectiveness outside narrowly defined environment. Microbial consortia may offer another approach and while promising in controlled environments, have been reported to sometimes fail to establish or function optimally in open-field conditions (Clagnan et al., 2024) whilst other studies demonstrate that microbial consortia, comprising multiple strains, offer superior benefits for soil health and plant productivity compared to single-strain inoculants, due to their enhanced functional resilience, ecological integration, and synergistic interactions (Soltero-Rios M et al., 2024).

Introduced microbial strains can interact with native soil communities in both beneficial and disruptive ways. Positive interactions may include synergistic nutrient cycling, enhanced plant growth, and suppression of pathogens through competitive exclusion. Negative outcomes may include competition with native microbiota which presents an additional barrier, as introduced strains may be outcompeted or inhibited, leading to poor persistence without repeated applications or environmental support. Also, the introduction of poorly characterized or non-native strains may disrupt native soil microbiota, potentially leading to ecological imbalances and reduced microbial diversity (Arora and Mishra, 2024). Regulatory and biosafety concerns, particularly regarding genetically modified or non-native strains also pose further challenges. Approval processes are often protracted and vary significantly across jurisdictions, impeding timely deployment. Moreover, field performance remains inconsistent and results obtained under laboratory or greenhouse conditions often do not translate to complex, real-world environments. There is growing interest in developing smart farming methodologies that integrates diverse technologies to improve entire agricultural systems (Imran Ortas, 2025; Waoo A et al., 2025; Hartman et al., 2018). A critical component of this approach should involve the development and targeted application of precision biofertiliers to improve soil health and enhance food productivity.
